# Novel models for predicting individualized outcomes in patients with advanced hepatocellular carcinoma receiving immunotherapy

**DOI:** 10.3389/fonc.2026.1756928

**Published:** 2026-06-01

**Authors:** Chao Chen, Yang Wang, Yan Zhao, Wenyan Cao, Ao Chen, Xiufeng Liu, Zhan Shi, Jie Shen

**Affiliations:** 1 Comprehensive Cancer Center, Nanjing Drum Tower Hospital Clinical College of Nanjing University of Chinese Medicine, Nanjing, China; 2 Department of Oncology, Jinling Hospital, Affiliated Hospital of Medical School, Nanjing University, Nanjing, China; 3 Jinling Hospital, Affiliated Hospital of Medical School, Nanjing University, Nanjing, China

**Keywords:** hepatocellular carcinoma, immunotherapy, nomogram, predictor, survival

## Abstract

**Background:**

We aim to develop a nomogram that can effectively differentiate target hepatocellular carcinoma (HCC) patients to receive immunotherapy and categorize their risk levels.

**Methods:**

This retrospective study analyzed 328 patients with HCC who had received anti-programmed cell death-1 (PD-1) drugs between January 2019 and December 2022. Univariate and multivariate Cox regression analyses were used to identify potential prognostic factors. The novel nomograms were then established based on the above independent predictors and assessed by Harrell’s concordance index (C-index), receiver operating characteristic (ROC) curves, calibration curves, and decision curve analyses (DCA). Kaplan-Meier curves were performed to estimate the progression-free survival (PFS) and overall survival (OS) based on risk scores.

**Results:**

Survival analyses identified the treatment sequence, disease progression with bone or lymph node, and Child-Pugh classification as prognostic factors for PFS and Barcelona Clinic Liver Cancer (BCLC) stage, Child-Pugh stage, ascites, Eastern Cooperative Oncology Group Performance Status (ECOG PS), surgery, disease progression with lymph node, and neutrophil-to-lymphocyte ratio (NLR) for OS. The PFS model had a C-index of 0.657 (0.609-0.705), matching validation set performance [0.657 (0.582-0.733)]. OS model C-indices were 0.787 (0.748-0.826) and 0.671 (0.606-0.736) for training and validation cohorts. Calibration plots and DCA curves indicated high accuracy and applicability. Significant differences in OS and PFS times were observed between low- and high-risk groups based on current nomogram points (*P* < 0.0001).

**Conclusions:**

The prognostic nomogram based on patients’ demographics and clinicopathological factors showed reliable efficacy in predicting survival benefits in intermediate and advanced-stage HCC patients following immunotherapy, aiding in individual decision-making.

## Introduction

Hepatocellular carcinoma (HCC) remains a major public health challenge worldwide, with a high incidence and increasing mortality (1). Approximately 70% of HCC patients are diagnosed with an intermediate or advanced stage, making them ineligible for surgical resection, liver transplantation, or local ablation (2). Systemic therapies, especially for immunotherapy-based treatment regimens, have shown promising outcomes in bolstering anti-tumor efficacy and improving survival (3, 4).

Immune checkpoint inhibitors (ICIs) are the most commonly used immunotherapy drugs, including anti-programmed death-1 (PD-1) antibody, anti-programmed death ligand-1 (PD-L1) antibody, and anti-cytotoxic T lymphocyte antigen 4 (CTLA4) antibody (5). Based on the IMbrave 150 study (6), atezolizumab (a PD-L1 inhibitor) in combination with bevacizumab (an anti-angiogenic agent) is recommended as the first-line treatment for patients with unresectable HCC. Similarly, phase III clinical trials of ORIENT-32 and CARES-310 further confirmed the encouraging efficacy and manageable adverse events with the combination of immunotherapy and targeted therapy (7, 8). Additionally, dual ICIs with durvalumab (an anti-PD-L1 antibody) plus tremelimumab (an anti-CTLA4 antibody) have also shown encouraging results (9). Furthermore, ICIs have also been recommended as the second-line treatment regimen for HCC patients (10–12).

PD-L1 expression, tumor mutational burden (TMB), and microsatellite status are reported to predict the effectiveness of immunotherapy in various cancers, including HCC (13–16). However, as a result of the low positive rates and limited frequency of surgical procedures and biopsies for HCC, the utilization of biomarkers that rely on pathological identification is constrained. Additionally, some peripheral blood markers have also been found to be associated with the tumor response to anti-PD-1 therapy (17–19). More recently, Zeng et al. established an artificial intelligence model to assess the expression of atezolizumab-bevacizumab response signature and further predict clinical benefits (20), but they have not been validated. Therefore, there was a critical need to investigate non-invasive and easily available biomarkers that are suitable for HCC patients receiving immunotherapy.

Recent nomogram-based studies have explored prognostic stratification in HCC using molecular signatures, as well as clinicopathologic models in patients with solid tumors treated with immunotherapy (21–23). However, many of these approaches rely on molecular assays or disease contexts that may limit their immediate applicability to routine management of advanced HCC. Therefore, a prognostic model based on readily available clinical variables may provide a practical tool for bedside risk assessment and real-world decision support. In this study, we investigated the association between clinicopathological characteristics and survival outcomes in patients with intermediate- and advanced-stage HCC receiving immunotherapy, identified prognostic factors associated with PFS and OS, and developed nomogram models to support individualized prognostic stratification and clinical management in this population.

## Methods

### Patients

Patients were included with the following criteria: diagnosed with HCC; underwent at least two cycles of immunotherapy; older than 18 years; had an Eastern Cooperative Oncology Group Performance Status (ECOG PS) of 0-2; had imaging evaluations before and after immunotherapy; and had adequate liver and renal function. Patients who met any of the criteria were excluded: received immunotherapy previously; underwent anti-PD-1 therapy less than two cycles; without computed tomography (CT) and/or magnetic resonance imaging (MRI) images; absence of baseline clinicopathological data; or discontinued follow-up. All procedures followed the Helsinki Declaration, and the study obtained approval from the hospital’s ethics committee (No. DZQH-KYLL-23-06). All patients involved in the study agreed with and signed the consent.

### Treatment and evaluation

All patients received ICIs as instructions, briefly, PD-1 inhibitors were administered intravenously to patients according to recommended doses every 2 or 3 weeks. Throughout the treatment period, CT and/or MRI were performed every 2 or 3 months to evaluate tumor response using the Response Evaluation Criteria in Solid Tumour (RECIST) (24) and immune RECISIT (iRECIST) (25). The primary study endpoints were progression-free survival (PFS) and overall survival (OS). PFS was defined as the duration from the start of ICIs to disease progression, and OS was defined as the interval between the initial treatment of ICIs and the death or the last follow-up (August 15th, 2023).

### Clinical data collection

Demographic and clinical characteristics involving age, gender, ECOG PS, hepatic virus infection, Child-Pugh stage, Barcelona Clinic Liver Cancer (BCLC) stage, metastatic sites, treatment procedures, and sites of disease progression were obtained from medical record system. Additionally, routine laboratory results, including of alpha-fetoprotein (AFP), white blood cell (WBC), hemoglobin, bilirubin, albumin, and lactate dehydrogenase (LDH) levels were incorporated into this study, and the inflammatory markers, such as platelet-to-lymphocyte ratio (PLR, platelet count/lymphocyte count) and neutrophil-to-lymphocyte ratio (NLR, neutrophil count/lymphocyte count) were calculated based on relevant data. Adverse effects were assessed using the Common Terminology Criteria of Adverse Events (CTCAE) version 5.0.

### Statistical analysis

All statistical analyses were performed using the R programming language version 4.3.1. A *P* value of less than 0.05 was deemed statistically significant for differences. Categorical variables were assessed using either the chi-squared test or Fisher’s exact test. Significant predictors were determined by univariate and multivariate Cox regression models, and all results were presented as hazard ratio (HR) with a 95% confidence interval (CI).

A nomogram was subsequently created according to the independent prognostic factors for the estimation of PFS and OS at 6, 12, and 24 months for HCC patients after immunotherapy. The discrimination of the nomogram was evaluated using Harrell’s concordance index (C-index), receiver operating characteristic (ROC) curve, as well as the area under the curve (AUC). Calibration plots were used to assess the accuracy. Additionally, decision curve analysis (DCA) was performed to evaluate the clinical utility of the predictive models by measuring the net benefits of the nomogram in guiding decision-making. Furthermore, the Kaplan-Meier method and the log-rank test were conducted to depict the differences in PFS and OS for patients in the high-risk or low-risk subgroups based on their individual risk scores.

## Results

### Baseline characteristics of the study cohort

**Figure 1:**
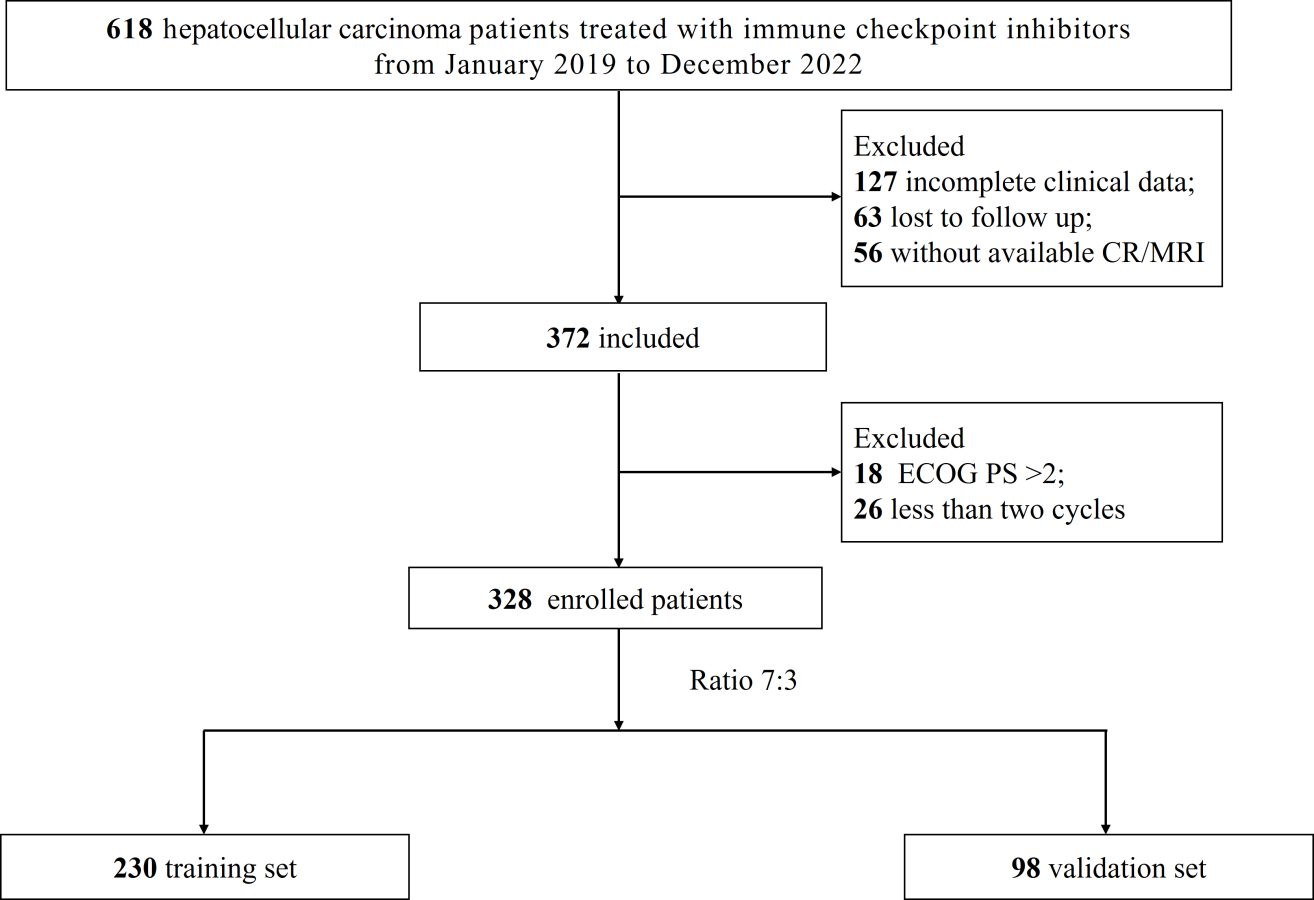
Patient flowchart. HCC, hepatocellular carcinoma; ICIs, immune checkpoint inhibitors; CT, computed tomography; MRI, magnetic resonance imaging; ECOG PS, Eastern Cooperative Oncology Group Performance Status; ROC, receiver operating characteristic; DCA, decision curve analyses.

From January 2019 to December 2022, an amount of 618 HCC patients received anti-PD-1 therapy, and finally 328 patients were enrolled in this study. The study design is displayed in (**Figure 1**). All patients were randomly assigned into a training cohort (n = 230) and a validation cohort (n = 98) in a ratio of 7:3, with similar durations of follow-up of 14.6 (Q1-Q3 5.03-26) and 12.2 (Q1-Q3 4-24.78) months, respectively. As shown in the **Table 1**, during the follow-up, there were 135 and 69 patients died in the training and validation set. The validation group had a higher proportion of patients with Barcelona Clinic Liver Cancer (BCLC)-C stage, lung metastases, and higher levels of lactate dehydrogenase (LDH) (> 246 U/L), and other variables did not differ significantly between the two groups. In total, 282 patients were aged 40–70 years, and the majority were male (86.59%). Of the patients, 296 had hepatitis B virus (HBV) infections. Cirrhosis was found in 260 patients (79.27%). 256 patients were classified as BCLC-C stage. 267 (81.40%) patients had intrahepatic metastasis, and lung (26.22%) and lymph node (25.30%) were the most common extrahepatic metastatic patterns. Patients treated with surgery, transarterial chemoembolization (TACE), and radiotherapy were 150, 212, and 96, respectively. The most commonly used PD-1 inhibitors were camrelizumab (63.11%) and sintilimab (15.24%). A total of 103 patients (31.40%) received tyrosine kinase inhibitors (TKIs) as initial treatment, while 97 patients (29.57%) received ICIs, and an additional 128 patients (39.02%) received both drugs simultaneously. The most common sites of disease progression were liver (78.96%) and lung (14.94%).

**Table 1:** Baseline characteristics.

Variables	Total (n = 328)	Training set (n = 230)	Validation set (n = 98)	*P*
Months, median (Q1, Q3)	14.2 (4.88, 25.55)	14.6 (5.03, 26)	12.2 (4, 24.78)	0.474
Status				0.060
Alive	124 (37.80)	95 (41.30)	29 (29.59)	
Dead	204 (62.20)	135 (58.70)	69 (70.41)	
Age (years)				0.066
<40	26 (7.93)	14 (6.09)	12 (12.24)	
40-70	282 (85.98)	199 (86.52)	83 (84.69)	
>70	20 (6.10)	17 (7.39)	3 (3.06)	
Gender				0.900
Male	284 (86.59)	200 (86.96)	84 (85.71)	
Female	44 (13.41)	30 (13.04)	14 (14.29)	
ECOG PS				1
0-1	250 (76.22)	175 (76.09)	75 (76.53)	
2	78 (23.78)	55 (23.91)	23 (23.47)	
HBV				0.666
No	32 (9.76)	24 (10.43)	8 (8.16)	
Yes	296 (90.24)	206 (89.57)	90 (91.84)	
HCV				0.516
No	317 (96.65)	221 (96.09)	96 (97.96)	
Yes	11 (3.35)	9 (3.91)	2 (2.04)	
Cirrhosis				0.808
No	68 (20.73)	49 (21.30)	19 (19.39)	
Yes	260 (79.27)	181 (78.70)	79 (80.61)	
Child-Pugh stage				0.863
A	258 (78.66)	182 (79.13)	76 (77.55)	
B	70 (21.34)	48 (20.87)	22 (22.45)	
BCLC Stage				**0.041**
B	72 (21.95)	58 (25.22)	14 (14.29)	
C	256 (78.05)	172 (74.78)	84 (85.71)	
Ascites				0.512
No	198 (60.37)	142 (61.74)	56 (57.14)	
Yes	130 (39.63)	88 (38.26)	42 (42.86)	
PVTT grade				0.631
<3	189 (57.62)	135 (58.70)	54 (55.10)	
≥3	139 (42.38)	95 (41.30)	44 (44.90)	
Probiotics				0.989
No	303 (92.38)	213 (92.61)	90 (91.84)	
Yes	25 (7.62)	17 (7.39)	8 (8.16)	
Metastatic sites				
Liver				0.481
No	61 (18.60)	40 (17.39)	21 (21.43)	
Yes	267 (81.40)	190 (82.61)	77 (78.57)	
Lung				**0.032**
No	242 (73.78)	178 (77.39)	64 (65.31)	
Yes	86 (26.22)	52 (22.61)	34 (34.69)	
Lymph node				0.637
No	245 (74.70)	174 (75.65)	71 (72.45)	
Yes	83 (25.30)	56 (24.35)	27 (27.55)	
Bone				0.666
No	296 (90.24)	206 (89.57)	90 (91.84)	
Yes	32 (9.76)	24 (10.43)	8 (8.16)	
Others				0.189
No	291 (88.72)	208 (90.43)	83 (84.69)	
Yes	37 (11.28)	22 (9.57)	15 (15.31)	
Therapy line				0.864
1	213 (64.94)	150 (65.22)	63 (64.29)	
2	73 (22.26)	52 (22.61)	21 (21.43)	
>2	42 (12.8)	28 (12.17)	14 (14.29)	
Prior treatment				
Surgery				0.575
No	178 (54.27)	122 (53.04)	56 (57.14)	
Yes	150 (45.73)	108 (46.96)	42 (42.86)	
Radiotherapy				0.961
No	232 (70.73)	162 (70.43)	70 (71.43)	
Yes	96 (29.27)	68 (29.57)	28 (28.57)	
TACE				1
No	116 (35.37)	81 (35.22)	35 (35.71)	
Yes	212 (64.63)	149 (64.78)	63 (64.29)	
Targeted therapy				0.631
No	139 (42.38)	95 (41.30)	44 (44.90)	
Yes	189 (57.62)	135 (58.70)	54 (55.10)	
Chemotherapy				0.347
No	288 (87.80)	205 (89.13)	83 (84.69)	
Yes	40 (12.20)	25 (10.87)	15 (15.31)	
TBP				0.371
No	140 (42.68)	94 (40.87)	46 (46.94)	
Yes	188 (57.32)	136 (59.13)	52 (53.06)	
Types of ICIs				0.763
Camrelizumab	207 (63.11)	145 (63.04)	62 (63.27)	
Nivolumab	16 (4.88)	10 (4.35)	6 (6.12)	
Tislelizumab	22 (6.71)	15 (6.52)	7 (7.14)	
Sintilimab	50 (15.24)	34 (14.78)	16 (16.33)	
Others	33 (10.06)	26 (11.30)	7 (7.14)	
Medicine sequence				0.371
Prior TKIs	103 (31.40)	67 (29.13)	36 (36.73)	
Prior ICIs	97 (29.57)	69 (30.00)	28 (28.57)	
Synchronous	128 (39.02)	94 (40.87)	34 (34.69)	
Sites of disease progression				
Liver				0.794
No	69 (21.04)	47 (20.43)	22 (22.45)	
Yes	259 (78.96)	183 (79.57)	76 (77.55)	
Lung				0.529
No	279 (85.06)	198 (86.09)	81 (82.65)	
Yes	49 (14.94)	32 (13.91)	17 (17.35)	
Lymph node				0.767
No	305 (92.99)	215 (93.48)	90 (91.84)	
Yes	23 (7.01)	15 (6.52)	8 (8.16)	
Bone				1
No	305 (92.99)	214 (93.04)	91 (92.86)	
Yes	23 (7.01)	16 (6.96)	7 (7.14)	
Others				0.954
No	300 (91.46)	211 (91.74)	89 (90.82)	
Yes	28 (8.54)	19 (8.26)	9 (9.18)	
Adverse events grade				0.629
≤2	278 (84.76)	193 (83.91)	85 (86.73)	
3/4	50 (15.24)	37 (16.09)	13 (13.27)	
Laboratory examination				
AFP (ng/mL)				0.657
≤10	14 (4.27)	9 (3.91)	5 (5.10)	
10-200	69 (21.04)	51 (22.17)	18 (18.37)	
>200	245 (74.70)	170 (73.91)	75 (76.53)	
WBC (×10^9/L)				0.561
<3.5	65 (19.82)	48 (20.87)	17 (17.35)	
≥3.5	263 (80.18)	182 (79.13)	81 (82.65)	
Hemoglobin (g/L)				0.096
<120	121 (36.89)	92 (40.00)	29 (29.59)	
≥120	207 (63.11)	138 (60.00)	69 (70.41)	
Bilirubin (μmol/L)				0.061
<20.5	217 (66.16)	160 (69.57)	57 (58.16)	
≥20.5	111 (33.84)	70 (30.43)	41 (41.84)	
Albumin (g/L)				0.657
<35	54 (16.46)	36 (15.65)	18 (18.37)	
≥35	274 (83.54)	194 (84.35)	80 (81.63)	
LDH (U/L)				**0.002**
<120	6 (1.83)	6 (2.61)	0 (0)	
120-246	186 (56.71)	142 (61.74)	44 (44.90)	
>246	136 (41.46)	82 (35.65)	54 (55.10)	
NLR				0.630
<2	43 (13.11)	32 (13.91)	11 (11.22)	
≥2	285 (86.89)	198 (86.09)	87 (88.78)	
PLR				0.242
<100	72 (21.95)	55 (23.91)	17 (17.35)	
≥100	256 (78.05)	175 (76.09)	81 (82.65)	

ECOG PS, Eastern Cooperative Oncology Group Performance Status; HBV, hepatitis B virus; HCV, hepatitis C virus; BCLC, Barcelona clinical liver cancer; PVTT, portal vein tumor thrombus; TACE, transarterial chemoembolization; TBP, treatment beyond progression; ICIs, immune checkpoint inhibitors; TKIs, tyrosine kinase inhibitors; AFP, alpha-fetoprotein; WBC, white blood cells; LDH, lactate dehydrogenase; NLR, neutrophil-to-lymphocyte ratio; PLR, platelet-to-lymphocyte ratio. Bold values indicate statistical significance (P < 0.05).

### Independent prognostic factors

To identify factors associated with PFS, we first performed univariate Cox regression analysis. BCLC stage, ascites, bone metastasis, targeted therapy, treatment sequence, sites of disease progression, and Child-Pugh stage were identified as candidate prognostic factors. In the multivariate Cox regression analysis, Child-Pugh stage remained independently associated with PFS (HR 2.122, 95% CI 1.369–3.290, P = 0.001). Prior immunotherapy was also an independent predictor of PFS (HR 0.498, 95% CI 0.320–0.777, P = 0.002). In addition, bone progression (HR 2.277, 95% CI 1.355–3.827, P = 0.002) and lymph node progression (LNP; HR 1.982, 95% CI 1.085–3.621, P = 0.026) were independently associated with poorer PFS in HCC patients receiving immunotherapy (**Table 2**). For OS, multivariate Cox regression analysis identified several independent prognostic factors. These included ECOG PS (HR 2.050, 95% CI 1.327–3.168, P = 0.001), Child-Pugh stage (HR 1.951, 95% CI 1.190–3.197, P = 0.008), BCLC stage (HR 3.866, 95% CI 2.314–6.458, P < 0.001), ascites (HR 2.208, 95% CI 1.429–3.411, P < 0.001), lymph node progression (LNP; HR 2.338, 95% CI 1.285–4.257, P = 0.005), and NLR (HR 2.042, 95% CI 1.138–3.662, P = 0.017). Prior surgery was associated with improved OS (HR 0.665, 95% CI 0.455–0.972, P = 0.035). These variables were incorporated into the OS prediction model (**Table 3**.

**Table 2:** Univariate and multivariate analyses for PFS.

Variables	Univariate			Multivariate		
	HR	95% CI	*P*	HR	95% CI	*P*
Age (years)						
<40	Ref					
40-70	0.836	0.472-1.482	0.540			
>70	0.545	0.232-1.277	0.162			
Gender						
Male	Ref					
Female	1.403	0.904-2.177	0.131			
ECOG PS						
0-1	Ref					
2	1.399	0.959-2.040	0.081			
HBV						
No	Ref					
Yes	1.174	0.675-2.039	0.570			
HCV						
No	Ref					
Yes	0.727	0.232-2.285	0.586			
Cirrhosis						
No	Ref					
Yes	0.844	0.579-1.229	0.376			
Child-Pugh stage						
A	Ref			Ref		
B	1.681	1.114-2.538	0.013	2.122	1.369-3.290	**0.001**
BCLC Stage						
B	Ref					
C	1.564	1.045-2.342	0.030	1.342	0.889-2.027	0.162
Ascites						
No	Ref					
Yes	1.370	0.975-1.925	0.070			
PVTT grade						
<3	Ref					
≥3	1.128	0.809-1.574	0.477			
Probiotics						
No	Ref					
Yes	0.747	0.430-1.298	0.301			
Metastatic sites						
Liver						
No	Ref					
Yes	1.204	0.776-1.867	0.408			
Lung						
No	Ref					
Yes	1.104	0.764-1.595	0.599			
Lymph node						
No	Ref					
Yes	1.434	0.994-2.068	0.054			
Bone						
No	Ref					
Yes	1.931	1.171-3.185	0.010	2.277	1.355-3.827	0.002
Others						
No	Ref					
Yes	1.410	0.877-2.266	0.156			
Prior treatment						
Surgery						
No	Ref					
Yes	0.746	0.537-1.037	0.081			
Radiotherapy						
No	Ref					
Yes	0.849	0.599-1.203	0.358			
TACE						
No	Ref					
Yes	1.226	0.863-1.743	0.255			
Targeted therapy						
No	Ref					
Yes	1.475	1.046-2.078	0.027	1.246	0.852–1.823	0.257
Chemotherapy						
No	Ref					
Yes	1.302	0.818-2.072	0.266			
TBP						
No	Ref					
Yes	0.811	0.580-1.134	0.220			
Types of ICIs						
Camrelizumab	Ref					
Nivolumab	1.659	0.903-3.047	0.103			
Tislelizumab	1.549	0.841-2.854	0.160			
Sintilimab	1.249	0.807-1.933	0.318			
Others	1.434	0.824-2.495	0.202			
Medicine sequence						
Prior TKIs	Ref			Ref		
Prior ICIs	0.582	0.378-0.896	0.014	0.498	0.320-0.777	**0.002**
Synchronous	1.067	0.735-1.549	0.733			
Sites of disease progression						
Liver						
No	Ref					
Yes	0.734	0.503-1.072	0.110			
Lung						
No	Ref					
Yes	1.261	0.836-1.902	0.268			
Lymph node						
No	Ref			Ref		
Yes	1.863	1.030-3.370	0.040	1.982	1.085-3.621	**0.026**
Bone						
No	Ref			Ref		
Yes	2.633	1.575-4.400	<0.001	2.277	1.355-3.827	**0.002**
Others						
No	Ref					
Yes	1.695	1.056-2.720	0.029	1.495	0.917-2.436	0.107
Adverse events grade						
≤2	Ref					
3/4	1.341	0.877-2.050	0.176			
Laboratory examination						
AFP (ng/mL)						
≤10	Ref					
10-200	1.006	0.422-2.400	0.989			
>200	1.000	0.439-2.280	0.999			
WBC (×10^9/L)						
<3.5	Ref					
≥3.5	1.187	0.785-1.795	0.417			
Hemoglobin (g/L)						
<120	Ref					
≥120	1.055	0.740-1.503	0.769			
Bilirubin (μmol/L)						
<20.5	Ref					
≥20.5	1.283	0.914-1.802	0.150			
Albumin (g/L)						
<35	Ref					
≥35	0.718	0.469-1.100	0.128			
LDH (U/L)						
<120	Ref					
120-246	2.752	0.383-19.795	0.315			
>246	3.349	0.464-24.191	0.231			
NLR						
<2	Ref					
≥2	1.196	0.752-1.901	0.449			
PLR						
<100	Ref					
≥100	1.216	0.834-1.774	0.310			

PFS, progression-free survival; ECOG PS, Eastern Cooperative Oncology Group Performance Status; HBV, hepatitis B virus; HCV, hepatitis C virus; BCLC, Barcelona clinical liver cancer; PVTT, portal vein tumor thrombus; TACE, transarterial chemoembolization; TBP, treatment beyond progression; ICIs, immune checkpoint inhibitors; TKIs, tyrosine kinase inhibitors; AFP, alpha-fetoprotein; WBC, white blood cells; LDH, lactate dehydrogenase; NLR, neutrophil-to-lymphocyte ratio; PLR, platelet-to-lymphocyte ratio; HR, hazard ratio; CI, confidence interval. Bold values indicate statistical significance (P < 0.05).

**Table 3:** Univariate and multivariate analyses for OS.

Variables	Univariate			Multivariate		
	HR	95% CI	*P*	HR	95% CI	*P*
Age (years)						
<40	Ref					
40-70	0.998	0.522-1.908	0.996			
>70	0.643	0.253-1.631	0.352			
Gender						
Male	Ref					
Female	1.698	1.055-2.734	0.029	1.345	0.821-2.204	0.238
ECOG PS						
0-1	Ref			Ref		
2	1.867	1.258-2.769	0.002	2.050	1.327-3.168	**0.001**
HBV						
No	Ref					
Yes	1.273	0.718-2.256	0.409			
HCV						
No	Ref					
Yes	1.006	0.411-2.461	0.989			
Cirrhosis						
No	Ref					
Yes	1.189	0.770-1.836	0.434			
Child-Pugh stage						
A	Ref			Ref		
B	3.921	2.658-5.785	<0.001	1.951	1.190-3.197	**0.008**
BCLC Stage						
B	Ref			Ref		
C	3.277	1.992-5.391	<0.001	3.866	2.314-6.458	**<0.001**
Ascites						
No	Ref			Ref		
Yes	2.754	1.945-3.901	<0.001	2.208	1.429-3.411	**<0.001**
PVTT grade						
<3	Ref					
≥3	2.038	1.451-2.863	<0.001	1.562	0.941-2.593	0.086
Probiotics						
No	Ref					
Yes	0.943	0.521-1.707	0.846			
Metastatic sites						
Liver						
No	Ref					
Yes	1.062	0.683-1.652	0.790			
Lung						
No	Ref					
Yes	1.201	0.817-1.767	0.351			
Lymph node						
No	Ref					
Yes	1.717	1.188-2.481	0.004	1.385	0.892–2.151	0.146
Bone						
No	Ref					
Yes	1.416	0.837-2.394	0.194			
Others						
No	Ref					
Yes	1.361	0.781-2.369	0.277			
Therapy line						
1	Ref					
2	1.058	0.698-1.602	0.791			
>2	1.227	0.737-2.043	0.431			
Prior treatment						
Surgery						
No	Ref			Ref		
Yes	0.492	0.347-0.698	<0.001	0.665	0.455-0.972	**0.035**
Radiotherapy						
No	Ref					
Yes	0.716	0.493-1.040	0.080			
TACE						
No	Ref					
Yes	0.641	0.452-0.910	0.013	0.792	0.548–1.145	0.216
Targeted therapy						
No	Ref					
Yes	1.132	0.798-1.606	0.488			
Chemotherapy						
No	Ref					
Yes	1.347	0.799-2.273	0.264			
TBP						
No	Ref					
Yes	0.507	0.358-0.719	<0.001	0.709	0.473-1.063	0.096
Types of ICIs						
Camrelizumab	Ref					
Nivolumab	0.770	0.335-1.774	0.540			
Tislelizumab	1.135	0.568-2.267	0.721			
Sintilimab	1.496	0.954-2.347	0.079			
Others	0.903	0.526-1.549	0.710			
Medicine sequence						
Prior TKIs	Ref					
Prior ICIs	0.637	0.401-1.012	0.056			
Synchronous	0.829	0.560-1.227	0.348			
Sites of disease progression						
Liver						
No	Ref					
Yes	0.838	0.564-1.247	0.385			
Lung						
No	Ref					
Yes	1.106	0.700-1.747	0.666			
Lymph node						
No	Ref			Ref		
Yes	1.797	1.032-3.129	0.038	2.338	1.285-4.257	**0.005**
Bone						
No	Ref					
Yes	1.633	0.937-2.846	0.084			
Others						
No	Ref					
Yes	1.531	0.879-2.668	0.133			
Adverse events grade						
≤2	Ref					
3/4	1.225	0.793-1.893	0.361			
Laboratory examination						
AFP (ng/mL)						
≤10	Ref					
10-200	0.786	0.302-2.043	0.621			
>200	1.049	0.426-2.581	0.917			
WBC (×10^9/L)						
<3.5	Ref					
≥3.5	1.049	0.687-1.602	0.824			
Hemoglobin (g/L)						
<120	Ref					
≥120	0.644	0.458-0.905	0.011	0.748	0.515-1.085	0.126
Bilirubin (μmol/L)						
<20.5	Ref					
≥20.5	1.418	0.991-2.030	0.056			
Albumin (g/L)						
<35	Ref					
≥35	0.505	0.326-0.782	0.002	0.725	0.468–1.123	0.149
LDH (U/L)						
<120	Ref					
120-246	1.679	0.529-5.330	0.380			
>246	2.527	0.788-8.102	0.119			
NLR						
<2	Ref			Ref		
≥2	1.949	1.099-3.455	0.022	2.042	1.138-3.662	**0.017**
PLR						
<100	Ref					
≥100	1.836	1.196-2.818	0.005	1.425	0.912–2.226	0.120

OS, overall survival; ECOG PS, Eastern Cooperative Oncology Group Performance Status; HBV, hepatitis B virus; HCV, hepatitis C virus; BCLC, Barcelona clinical liver cancer; PVTT, portal vein tumor thrombus; TACE, transarterial chemoembolization; TBP, treatment beyond progression; ICIs, immune checkpoint inhibitors; TKIs, tyrosine kinase inhibitors; AFP, alpha-fetoprotein; WBC, white blood cells; LDH, lactate dehydrogenase; NLR, neutrophil-to-lymphocyte ratio; PLR, platelet-to-lymphocyte ratio; HR, hazard ratio; CI, confidence interval. Bold values indicate statistical significance (P < 0.05).

### Nomogram construction and validation

**Figure 2:**
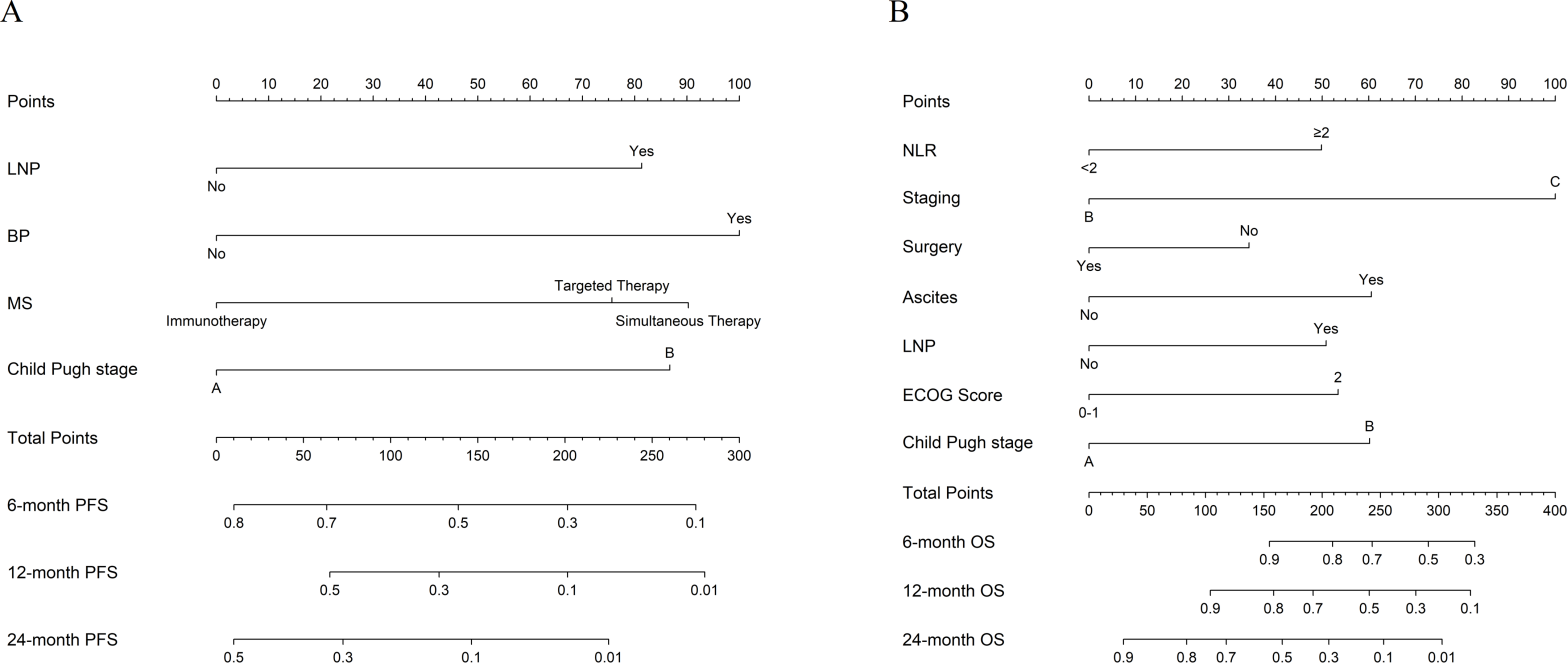
Nomograms for predicting 6-, 12-, and 24-month PFS **(A)** and OS **(B)** in HCC patients receiving immunotherapy. PFS, progression-free survival; OS, overall survival; LNP, progression of lymph node; BP, progression of bone; MS, medicine sequence; ECOG, Eastern Cooperative Oncology Group; NLR, neutrophil-to-lymphocyte ratio.

**Figure 3:**
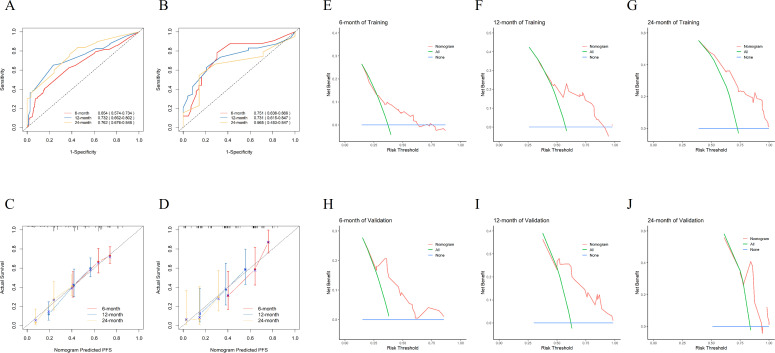
PFS prediction model. Time-dependent ROC curves of the nomogram to predict the 6-, 12-, and 24-month PFS in the training set **(A)** and the validation set **(B)**. The calibration curves predict the 6-, 12-, and 24-month PFS in the training set **(C)** and the validation set **(D)**. DCA curves for predicting the 6-, 12-, and 24-month PFS in the training set **(E-G)** and the validation set **(H-J)**. PFS, progression-free survival; ROC, receiver operating characteristic; DCA, decision curve analyses.

Based on the multivariate analysis results, we built a novel nomogram for predicting the PFS with the four prognostic factors, as shown in **Figure 2A**, an increased score, derived from the aggregate of the designated points for each prognostic factor within the nomogram, was associated with a reduced likelihood of survival within the 6, 12, or 24-month period. In terms of distinguishing ability, the C-index and 95% CI for the training dataset were 0.657 (0.609-0.705), and similar results were found in the validation dataset with 0.657 (0.582-0.733). The AUC values for 6-, 12-, and 24-month PFS were 0.654 (0.574-0.734), 0.732 (0.662-0.802), and 0.762 (0.676-0.848) in the training set, and 0.751 (0.636-0.866), 0.731 (0.615-0.847), and 0.665 (0.483-0.847) in the validation set, respectively (**Figures 3A, B**). The calibration plots depicted a good performance at the accuracy of the nomogram for predicting PFS (**Figures 3C, D**). Furthermore, the DCA curves for the model showed well clinical utility (**Figures 3E-J**).

**Figure 4:**
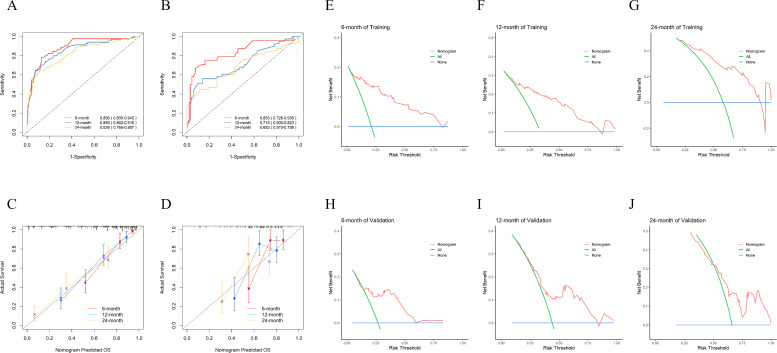
OS prediction model. Time-dependent ROC curves of the nomogram to predict the 6-, 12-, and 24-month OS in the training set **(A)** and the validation set **(B)**. The calibration curves predict the 6-, 12-, and 24-month OS in the training set **(C)** and the validation set **(D)**. DCA curves for predicting the 6-, 12-, and 24-month OS in the training set **(E-G)** and the validation set **(H-J)**. OS, overall survival; ROC, receiver operating characteristic; DCA, decision curve analyses.

In addition, we developed a nomogram for OS by assigning a specific weighted score to each individual prognostic parameter obtained from the multivariate Cox model, for example, patients who display an NLR of 2 or higher, categorized under the BCLC stage C, accompanied by the presence of ascites and lymph node progression (LNP), with an ECOG PS of 2, classified as Child-Pugh stage C, and who had not undergone surgical intervention, exhibit significantly inferior OS outcomes (**Figure 2B**). The C-index for predicting the OS were 0.787 (95% CI 0.748-0.826) and 0.671 (95% CI 0.606-0.736) in the training and validation set, respectively, which suggests a good discriminatory ability of the current nomogram. The ROC analysis demonstrated a 6-month AUC of 0.890 (0.835-0.945), 12-month AUC of 0.859 (0.802-0.916), and 24-month AUC of 0.826 (0.766-0.887) in the training set, respectively, and that of the validation cohort were 0.833 (0.728-0.939), 0.715 (0.606-0.823), and 0.683 (0.570-0.795) (**Figures 4A, B**). Moreover, the calibration plots indicated a high level of concordance between the observed and nomogram-predicted OS at various time intervals (**Figures 4C, D**). Finally, we evaluated the effectiveness by the DCA and identified a positive predictive ability of the nomogram (**Figures 4E-J**). This finding highlights the potential practical value of the model in making informed decisions and improving patients’ outcomes.

### Risk stratification based on the nomogram

**Figure 5:**
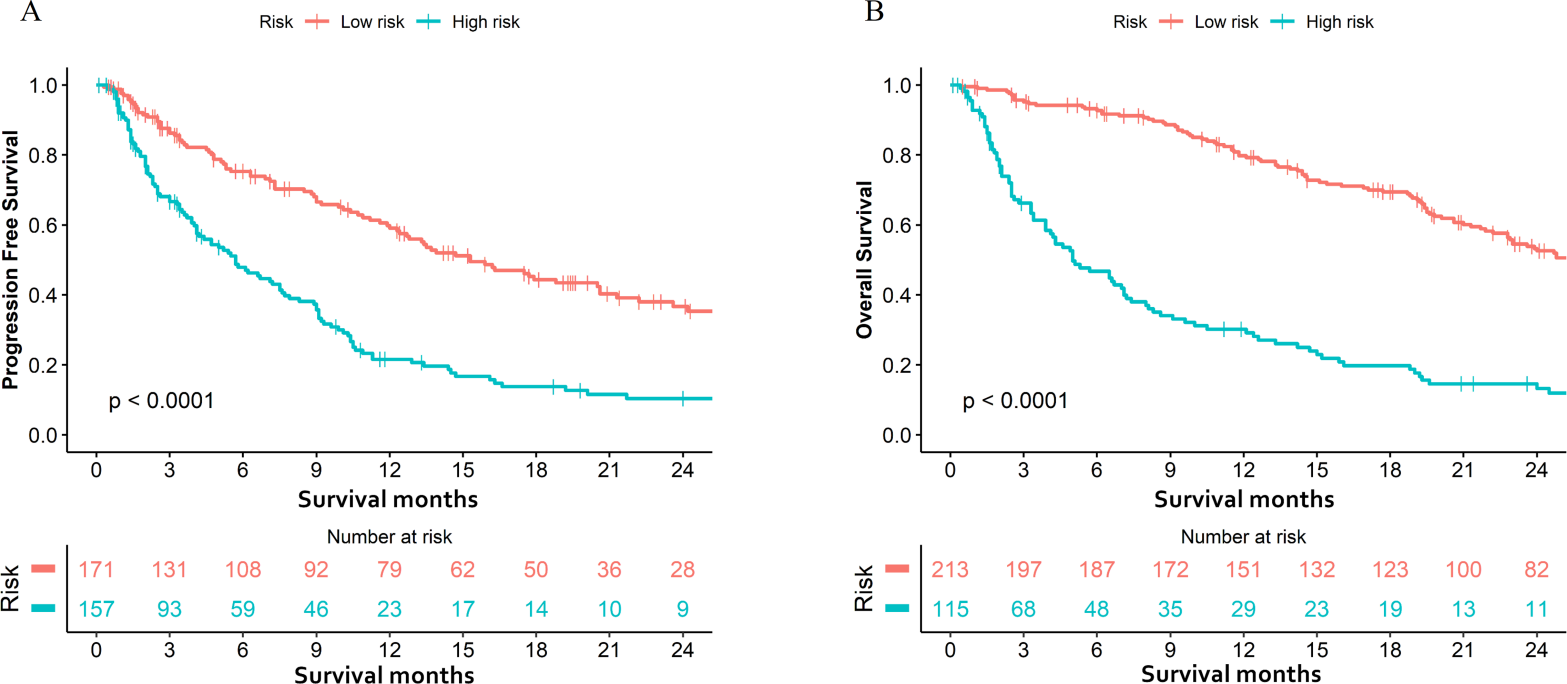
Kaplan-Meier curves for the PFS **(A)** and OS **(B)** among low- and high-risk HCC patients. Abbreviations: PFS, progression-free survival; OS, overall survival; HCC, hepatocellular carcinoma.

To further validate the predictive ability of the nomogram, we performed the subgroup analyses based on the previous prognostic factors using the following formula for PFS: risk score = (0.684 × LNP) + (0.823 × progression of bone) - (0.696 × medicine sequence) + (0.752 × Child-Pugh stage). As indicated by the **Figure 5A**, high-risk patients (>129.390) demonstrated a distinctly unfavorable PFS in comparison to those in the low-risk group (≤129.390) (1-year PFS rate: 14.6% vs. 46.2%, 2-year PFS rate: 5.7% vs. 16.4%, *P* < 0.0001), and we have designed an online web-based calculator (https://online-app.shinyapps.io/DynNomapp_PFS/) to help us more effectively assess each patient’s PFS. Additionally, the risk score for OS equals (0.6890 × NLR) + (1.3818 × Staging) - (0.4744 × Surgery) + (0.8365 × Ascites) + (0.7024 × LNP) + (0.7380 × ECOG PS) + (0.8315 × Child-Pugh stage). The Kaplan-Meier OS curves also demonstrated great discrimination among the high-risk (> 238.321) and low-risk (≤ 238.321) groups (1-year OS rate: 25.2% vs. 70.9%, 2-year OS rate: 9.6% vs. 38.5%, *P* < 0.0001) (**Figure 5B**). The corresponding webpage prediction tool for the nomogram of our study is located at https://online-app.shinyapps.io/DynNomapp_OS/. These results indicate that the nomogram model was able to differentiate intermediate and advanced-stage HCC patients who could potentially benefit from immunotherapy.

## Discussion

It is well known that immunotherapy has achieved significant advancements and played pivotal roles in the management of various malignancies in recent years (26, 27). However, the exorbitant drug costs, the occurrence of potential immune-related adverse events, and the uncertain effectiveness have led to some patients failing to receive immunotherapy. Therefore, it is imperative to establish and identify valuable prognostic markers for cancer patients. In the current study, we successfully developed a nomogram according to the baseline clinicopathological characteristics and therapeutic process that can predict survival benefits from immunotherapy for patients with HCC.

The prognostic model that we built based on a large population demonstrated a great ability to stratify the risk of intermediate and advanced-stage HCC patients following immunotherapy, which can be utilized to identify candidates for immunotherapy, determine appropriate treatment allocation, and especially to predict long-term survival benefits. Patients who are categorized as being at high risk via the nomogram are forecasted to have a poorer response, and we suggest that these individuals should undergo more intensive treatment and evaluation. Currently, most prognostic models for HCC patients are based on gene or RNA signatures, here we develop this nomogram by collecting and analyzing clinical datasets, which is more convenient and cost-effective. More importantly, this is the first report, to our knowledge, to predict both PFS and OS by assessing a great many factors in patients with intermediate and advanced-stage HCC.

Except for immunotherapy, targeted therapy is also a predominant treatment modality for cancer patients, but the optimal treatment sequence has been unclear. In 2021, Matsumoto et al. demonstrated that anti-angiogenic agents should be given concomitantly or following ICIs to achieve better clinical benefits for advanced non-small cell lung cancer (NSCLC) (28). Recently, the randomized phase III DREAMseq trial elucidated that BRAF V600-mutant metastatic melanoma patients treated with immunotherapy initially led to better 2-year OS (71.8% vs. 51.5%) and durable responses than those commencing with dual v-RAF murine sarcoma viral oncogene homolog B (BRAF)/mitogen-activated protein kinase kinase (MEK) inhibitors (29), favorable results were also reported by the SECOMBIT trial (30). Nevertheless, a phase II study conducted in advanced NSCLC patients with epidermal growth factor receptor (EGFR) mutation and PD-L1 positive expression showed unsatisfactory tumor response and serious adverse events with the treatment of immunotherapy and subsequent the EGFR-TKI therapy (31). Similarly, Lin and colleagues found that NSCLC patients administered ICI before TKI might lead to increased hepatotoxicity (32). In terms of HCC, anti-PD-1 therapy was reported to enhance the efficacy of sorafenib (a multikinase inhibitor) by increasing CD8^+^ T-cell infiltration (33). In the present study, 103 patients received initial treatment with TKIs, 97 had ICIs as initial therapy, and another 128 patients administrated the two drugs synchronously. By performing the Cox regression analyses, we observed that prior immunotherapy resulted in better OS, which suggests that sequential ICIs and targeted therapy may be beneficial for HCC patients, and further prospective trials are needed to determine the preferred treatment sequence to achieve the best clinical effects.

Inflammation is recognized as an important contributor to the development and progression of cancer, and various inflammatory factors have been reported to be related to the efficacy and safety of antitumor therapy (34, 35). Jia et al. demonstrated that peripheral blood parameters could predict the survival of advanced HCC patients treated with immunotherapy (36). In which, NLR, a well-acknowledged inflammatory biomarker, has been found to be an independent predictor for the treatment of HCC, including surgery, TACE, and targeted therapy (37). In 2022, a phase II study reported that lower NLR was positively associated with the response to PD-1 inhibitors among HCC patients (38). As shown in the Results, the cut-off value of NLR in our enrolled patients was 2, and 43 (13.11%) of them had a lower NLR. According to the univariate and multivariate Cox regression analyses, it was negatively related to the OS for HCC patients receiving immunotherapy (NLR < 2 vs. ≥ 2, HR 2.042, 95% CI 1.138-3.662, *P* = 0.017), whereas no significant difference was observed in PFS.

The lymph node is a common extrahepatic metastatic site in HCC, with reported incidence varying from 1.2% to 15.3% (39, 40). In our study, 83 (25.3%) patients presented with LNP, secondary only to lung metastasis (26.2%). The higher incidence of LNM observed in our study might be attributed to the high rate of BCLC-C stage (78.05%) among all enrolled patients. In terms of poorer survival for patients with LNM, the treatment for lymph nodes remains controversial in operable HCC patients, with some researchers finding regional lymphadenectomy to be safe and effective (41), while others reported that lymph node dissection failed to improve the prognosis, but increased surgery complexity and postoperative complications (42). For advanced-stage patients, locoregional therapies such as radiotherapy (43), radiofrequency ablation (RFA) (44), and TACE (45) were recommended. In our study, LMN led to poorer OS (HR 1.717, 95% CI 1.188-2.481, *P* = 0.004), but not PFS in HCC patients receiving immunotherapy, which was consistent with previous reports (46). During the treatment, 23 (7.01%) patients had disease progression with lymph node involvement, which was confirmed to be an independent prognostic factor, associated with poorer PFS (HR 1.982, 95% CI 1.085-3.621, *P* = 0.026) and OS (HR 2.338, 95% CI 1.285-4.257, *P* = 0.005) compared to those without lymph node progression. These results indicate that we should pay more attention to the status of lymph node progression, especially for patients who did not undergo lymphadenectomy during the surgical procedure.

The Child-Pugh classification is the most commonly used model to evaluate liver function in patients with HCC, which consists of the scoring of hepatic encephalopathy and ascites, serum albumin and bilirubin levels, and prothrombin time. It has become a prognostic indicator for HCC patients receiving liver resection, radiotherapy, TACE, RFA, and targeted therapy (47–51). Additionally, Zhang and colleagues identified the Child-Pugh score as a risk factor for hyperprogressive disease in HCC patients treated with anti-PD-1 antibodies (52). Recent research has also reported that patients with advanced HCC classified as Child-Pugh B have a lower treatment response, higher mortality risk, and poorer survival outcomes following ICI treatment (53). In this study, we found that patients with Child-Pugh A had superior PFS (HR 2.112, 95% CI 1.369-3.290, *P* = 0.001) and OS (HR 1.951, 95% CI 1.190-3.197, *P* = 0.008) compared to those with Child-Pugh B. These findings highlight the importance of monitoring liver function at the initiation of immunotherapy.

Several limitations should be addressed in our study. First, it is important to note that this was a retrospective study, which may have introduced selection bias. Second, certain clinical variables, such as the history of alcohol consumption, were not included in the analysis. Third, the more objective model of albumin-bilirubin (ALBI) grade may be advantageous for evaluating liver function. Finally, we did not perform external validation analyses.

In conclusion, we established a simple and applicable model that predicted the prognosis in HCC patients treated with immunotherapy, and it is essential to validate the current nomogram through independent external studies in the future.

## Data Availability

The raw data supporting the conclusions of this article will be made available by the authors, without undue reservation.
